# Synthesis of a Novel Chitosan/Basil Oil Blend and Development of Novel Low Density Poly Ethylene/Chitosan/Basil Oil Active Packaging Films Following a Melt-Extrusion Process for Enhancing Chicken Breast Fillets Shelf-Life

**DOI:** 10.3390/molecules26061585

**Published:** 2021-03-13

**Authors:** Aris E. Giannakas, Constantinos E. Salmas, Areti Leontiou, Maria Baikousi, Dimitrios Moschovas, Georgios Asimakopoulos, Nikolaos E. Zafeiropoulos, Apostolos Avgeropoulos

**Affiliations:** 1 Department of Food Science and Technology, University of Patras, 30100 Agrinio, Greece; 2 Department of Material Sci. & Engineering, University of Ioannina, 45110 Ioannina, Greece; mariabaikousi@gmail.com (M.B.); dmoschov@uoi.gr (D.M.); asimakopoulos.geo@gmail.com (G.A.); nzafirop@uoi.gr (N.E.Z.); aavger@uoi.gr (A.A.); 3 Department of Business Administration of Food and Agricultural Enterprises, University of Patras, 30100 Agrinio, Greece; aleontiu@upatras.gr

**Keywords:** low density poly-ethylene, chitosan, basil oil, active packaging, barrier properties, lipid oxidation, shelf life

## Abstract

An innovative process for the adsorption of the hydrophobic Basil-Oil (BO) into the hydrophilic food byproduct chitosan (CS) and the development of an advanced low-density polyethylene/chitosan/basil-oil (LDPE/CS_BO) active packaging film was investigated in this work. The idea of this study was the use of the BO as both a bioactive agent and a compatibilizer. The CS was modified to a CS_BO hydrophobic blend via a green evaporation/adsorption process. This blend was incorporated directly in the LDPE to produce films with advanced properties. All the obtained composite films exhibited improved packaging properties. The film with 10% CS_BO content exhibited the best packaging properties, i.e., 33.0% higher tensile stress, 31.0% higher water barrier, 54.3% higher oxygen barrier, and 12.3% higher antioxidant activity values compared to the corresponding values of the LDPE films. The lipid oxidation values of chicken breast fillets which were packaged under vacuum using this film were measured after seven and after fourteen days of storage. These values were found to be lower by around 41% and 45%, respectively, compared with the corresponding lipid oxidation values of pure LDPE film.

## 1. Introduction

The incorporation of biodegradable raw materials for the development of improved packaging, resulted from the global trend towards a cyclic economy and production techniques which exhibit positive environmental fingerprint [1,2]. In this direction, the use of chitosan (CS) as an alternative to the synthetic polymers, provides promising results in active packaging issues. CS is a linear polysaccharide produced by treating chitin. Chitin mainly comes from crustaceans’ shells, which is a food byproduct. The CS based films exhibited great potential for use as packaging materials with biodegradability, nontoxicity, antioxidant and antimicrobial activity [3]. 

Although CS cannot be used directly in industrial extrusion molding processes, CS based materials have been recently reported as versatile and cost-competitive materials for various industrial applications [4,5,6,7]. To overcome the obstacles of this raw material use for such applications, the co-extrusion of the CS with synthetic polymers such as LDPE is proposed by researchers [8,9,10,11,12,13]. The blending of the CS with the LDPE requires a compatibilizer such as co-maleic anhydride [9]. Park et al. [10] prepared LDPE/CS blends with low CS concentration, i.e., up to 8%, by using lactic acid as a compatibilizer. The incorporation of CS to LDPE decreased the tensile strength as well as both the water and oxygen barrier. When the obtained LDPE/CS films were used for the storage of fresh red meat, microorganisms on the meat surface were not inhibited, but a significant extension of red color shelf-life was observed in refrigerated sliced red meats. Reesha et al. [11] developed LDPE/CS antimicrobial packaging films with also low CS concentrations, i.e., up to 5%, by using maleic anhydride grafted LDPE (LDPE-g-MAH) as a compatible agent. In this case the CS incorporation to LDPE films decreased tensile strength and water barrier, but at the same time the oxygen barrier was increased. Analysis of storage quality indexes such as peroxide value, free fatty acid, total volatile base nitrogen, and aerobic plate count, revealed a good antibacterial behavior and an extension of shelf life of Tilapia for the LDPE/CS composite films compared to the pure LDPE films. Wang et al. [13] developed LDPE/CS blends by using LDPE-g-MAH as a compatibilizer. The CS concentration in these films was up to 10%. Results indicated that by increasing the CS concentration the tensile strength and the water barrier decreased, while oxygen barrier increased. Recently, Kusumastuti et al. [9] prepared LDPE/CS blends with high CS concentration, i.e., up to 40%, by adding maleic anhydride (MA) and tert-butyl peroxybenzoate (TBPB) as a compatibilizer and initiator respectively. By increasing the CS concentration, the results showed an increase of tensile stress and biodegradability of the obtained LDPE/CS films but no data on oxygen/water permeation were reported. Thus, in general, the use of compatibilizers improve the dispersion of CS into LDPE. Nevertheless, the LDPE/CS active films still exhibited reduced tensile and water barrier properties, which are crucial for LDPE packaging films.

Nowadays, the trend in the food industry is to develop active packaging films with non-synthetic antioxidants such as butyl-hydroxytoluene (BHT), butyl-hydroxyanisole (BHA), tert-butyl hydroxyquinone (TBHQ). The use of such synthetic antioxidants raises safety concerns for consumer health. The last global effort is to replace these synthetic antioxidants with natural antioxidants such as essential oils and natural extracts [14,15]. Essential oils (EO) which were extracted from plants, have been used as seasoning agents in foods and beverages for centuries [16]. Basil (*Ocimum basilicum* L.) is a popular culinary herb, and its leaves contain essential oils which potentially could be used for applications in food products [17]. Basil essential oil (BO) consists of more than 30 compounds, but the major components of this oil are estragole [18] and eugenol. The last one causes the strong clove scent of sweet basil [18,19]. It has been proved that BO exhibits antimicrobial effect against different bacteria and different fungi [20].

In the present work, the development, characterization, and packaging performance of a potentially active, packaging film is studied. This active packaging film consists of three materials: (1) the LDPE which is one of the most widely used polymers for flexible active packaging films, (2) the food byproduct CS which is a biodegradable biopolymer with antioxidant and antimicrobial activities, and which potentially could be used as packaging material, and (3) the extracted oil BO which exhibits antioxidant properties and antimicrobial effect against different bacteria. The main innovation in the current work is the use of BO as both an antioxidant agent and a compatibilizer. More specifically, this study aims to the development of a final LDPE/CS_BO active film without any loss in tensile and with higher water/oxygen barrier properties compared to the relevant properties of pure LDPE film. Basil oil has been previously used as an active agent in LDPE [21] films and in LDPE-based blends [22] but to the best of our knowledge, this is the first time used as both an active agent and as a compatibilizer. For this reason, BO was firstly adsorbed into CS via a facile and green method, and a CS_BO blend was produced. Then, the hydrophobic CS_BO blend was incorporated into the LDPE chains in four different wt%. concentrations (i.e., 5, 10, 20, and 30 wt%.) via an one-step extrusion molding process. All the LDPE/CS_BO active packaging films and the produced CS_BO blend characterized via various characterization techniques and the packaging performance was also investigated. The film with the highest water/oxygen barrier, improved tensile properties, and antioxidant activity was further tested as an active packaging film for chicken breast fillets packaging under vacuum. The lipid oxidation value of these fillets is determined as a final validation parameter for packaging using these films.

## 2. Results

### 2.1. XRD Analysis

**Figure 1:**
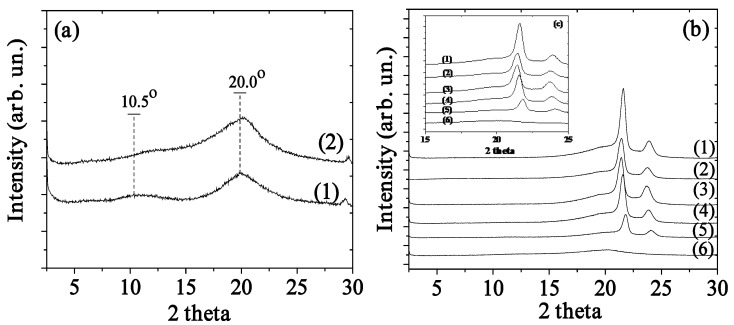
(**a**) XRD plots of (1) raw chitosan (CS) and (2) chitosan/basil-oil (CS_BO) powders. (**b**) and inset (**c**) XRD plots of (1) neat low-density polyethylene (LDPE), (2) LDPE/CS_BO5, (3) LDPE/CS_BO10, (4) LDPE/CS_BO20, (5) LDPE/CS_BO30, and (6) CS_BO powder.

According to previous literature reports [23,24], XRD plots of the pure CS and CS_BO samples (see Figure 1) exhibit two broad peaks at 2θ = 10.5° and 2θ = 20.0°. These peaks correspond to a hydrated crystalline structure and an amorphous CS, respectively [24]. XRD plots of the CS_BO hybrid show a clear shift of both CS peaks to higher angles. This peak shift indicates the interaction between CS chains and BO molecules during the process of the CS_BO blend development [8].

As it is obvious from all XRD plots of LDPE/CS_BO composite films (see Figure 1), by increasing the CS_BO content, the characteristic peak of the CS at around 20° increases, and the LDPE’s characteristic peaks shift to higher angles. Both observations indicate the effective blending of LDPE chains with CS_BO blend.

### 2.2. FTIR Results

**Figure 2:**
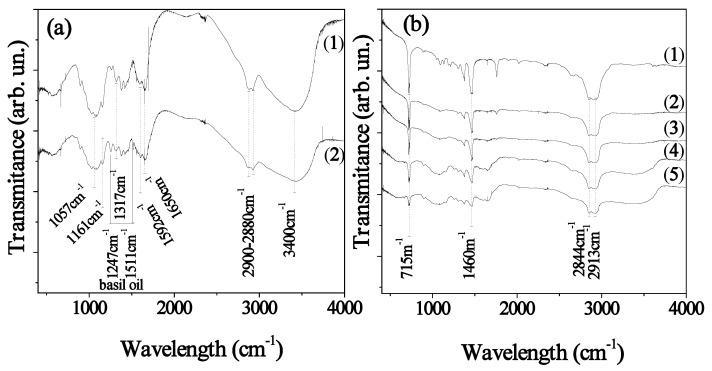
(**a**) FTIR spectra of (1) neat CS, and (2) CS_BO hybrid blend, (**b**) FTIR spectra of (1) neat LDPE, (2) LDPE/CS_BO5, (3) LDPE/CS_BO10, (4) LDPE/CS_BO20, and LDPE/CS_BO30 films.

Figure 2 depicts the FTIR spectra of the pure CS, of the modified CS_BO hybrid blend, and of all the LDPE/CS_BO films. FTIR spectra of the CS exhibit three main areas: (i) A broad asymmetric band between 3400 and 2500 cm^−1^. This band includes the CH stretching modes at around 2900 and 2880 cm^−1^, and the overlapped OH and NH stretching vibrations at higher wavenumbers (approximately 3400 cm^−1^); (ii) an area between 1700 and 1200 cm^−1^ which is characteristic of the amide groups; (iii) a strong absorption area between 1200 and 800 cm^−1^ which is characteristic of the CS saccharide structure [8,10,11,12,13].

The peaks at 1650 cm^−1^ and at 1590 cm^−1^ correspond to the stretching vibrations of the amide I (ν (C = O)) and the amide II (ν (NH_2_ in NHCOCH_3_)) groups. The peak at 1317 cm^−1^ corresponds to the bending vibration of the amide III group (δ (C-H)) in pyranose ring characteristic vibrations. The peak at 1161 cm^−1^ is attributed to the beta glycosidic bond between carbon 1 and carbon 4 of the CS. The peak at 1051 cm^−1^ can be associated with the COC stretching of the glucopyranoside ring. Finally, peaks at 1420 cm^−1^ and 1380 cm^−1^ represent the deformation bands of CH_2_ and CH_3_ [8,10,11,12,13]. After the incorporation of BO with the CS powder (see Figure 2a line (2)) two main peaks of BO [25] at approx. 1511 and 1547 cm^−1^ obtained. These peaks reveal the adsorption of BO molecules into CS chains. Moreover, the significant reduction of the CS_BO spectra compared to the CS spectra suggests a hydrogen bonding formation between BO components and CS chains [8,26].

In all FTIR plots of LDPE/CS_BO films, LDPE’s characteristic peaks are observed [8,10,11,12,27]. LDPE’s –CH_3_ asymmetric stretching, –CH_2_ wagging, and -CH_2_ rocking, correspond to peaks at 1460 and 715 cm^−1^, while the LDPE’s –CH_2_ symmetric stretching peaks are at 2913 and 2844 cm^−1^. It is also evident from all the FTIR plots of the LDPE/CS_BO films that the characteristic peaks of the CS exist in the range of 1900–1400 cm^−1^ and 3800–3200 cm^−1^. As the CS content increases, the detected LDPE bands decrease, and the CS bands enhance. This fact indicates the effective blending of the CS_BO with the LDPE. According to theory and previous reports [8,27], the interactions between chemical groups of dissimilar polymers could cause a position shift of peaks of the participating groups. In the present work, this kind of behavior is not observed for specific peaks. This result indicates that the preparation procedure which was chosen for the development of CS_BO blend and the modification of the CS with BO led to a hydrophobic CS_BO hybrid blend, which can easily mixed with the LDPE chains in short processing time (5 min, see Table 3) and without the addition of a compatibilizer.

### 2.3. Thermogravimetric (TG)/Differential Thermal Analysis (DTA) Results

**Figure 3:**
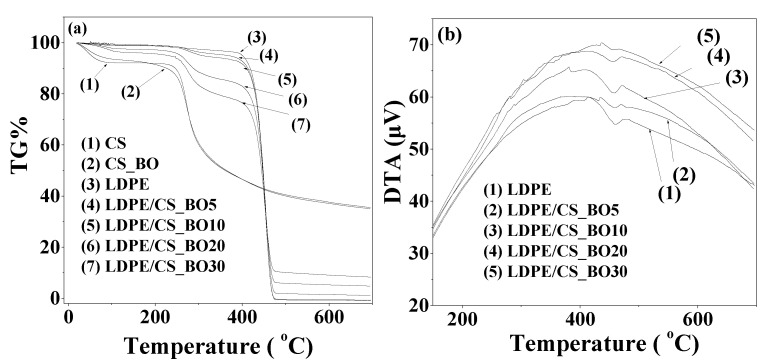
(**a**) Thermogravimetric (TG) plots of the pure CS and of the CS_BO blend as well as of all the obtained LDPE/CS_BO films, (**b**) differential thermal analysis (DTA) plots of all the obtained LDPE/CS_BO films.

Figure 3 shows the TG plots for the neat CS, CS_BO blend, and for all the LDPE/CS_BO blends. Both the pure CS and the CS_BO blend exhibit two weight loss steps in the TG plots. The first weight loss step which starts at around 100 °C and ends at around 200 °C occurs due to the evaporation of the adsorbed moisture. The second weight-loss step, which is the main, starts at approx. 230 °C and ends at approx. 550 °C. This is assigned to the decomposition of the CS chains [28]. It is evident from Figure 2a that the water weight loss step for the CS_BO hybrid blend, which occurs at around 100–120 °C, is lower than the corresponding water weight loss step of the pure CS. This result indicates that the hydrophobic CS_BO blend adsorbs less water than the hydrophilic pure CS. Furthermore, the decomposition weight loss step for the CS_BO blend starts at a temperature lower than this of the pure CS.

This result indicates the decrease of the thermal stability of the hydrophobically modified CS_BO blends as it is compared to the thermal stability of the pure CS [8]. The TG plot of the pure LDPE (see Figure 3a line 3) exhibits one weight loss step, which starts at around 395 °C [27]. The decomposition pathway of the material derived by incorporating the CS_BO blend with the LDPE chains is more complex than this of the pure of the pure LDPE and exhibits two steps instead of one [29]. By increasing the CS_BO content, these two weight loss decomposition steps of the TG plot, are shifted in lower temperatures. This shift is better shown in the DTA plots of the LDPE/CS_BO composite films (see Figure 2b). Thus, by increasing the CS_BO content, while the degradation temperature peak for LDPE is at 464.6 °C, the degradation temperature peak for LDPE/CS_BO5, LDPE/CS_BO10, LDPE/CS_BO20, and LDPE/CS_BO30 composites decreases at 459, 457 455, and 451 °C respectively. As it was reported recently [9], the decrease in degradation temperature of the LDPE/CS_BO blends by increasing the CS concentration, is a thermal behavior similar with this exhibited by the LDPE/CS blends.

### 2.4. DSC Results

**Figure 4:**
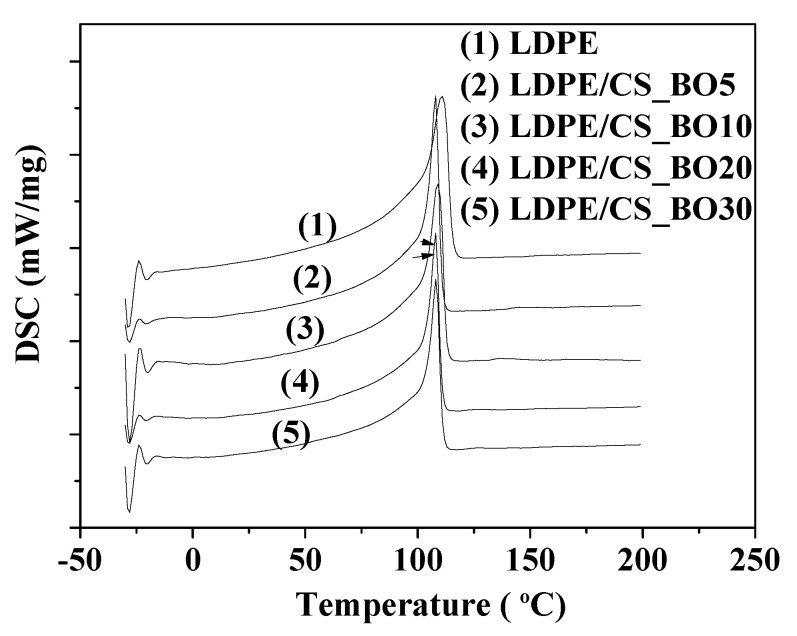
DSC curves of pure LDPE and LDPE/CS_BO composites.

Figure 4 presents the DSC curves of the LDPE/CS_BO films. The calculated characteristic temperatures and the fusion enthalpies are listed in Table 1.

The LDPE exhibits a melting temperature peak at around 111 °C, while the composites exhibit a melting temperature peak at around 107–109 °C. A decrease in melting temperature value of the LDPE/CS blends is also reported by Kusumastuti et al. [9]. Moreover, the LDPE shows a fusion enthalpy value of 113.5 J/g while the composites show fusion enthalpy values in the range 79–104 J/g. The LDPE/CS_BO10 sample exhibits the higher enthalpy value as it is compared to the corresponding values of the other composites (i.e., 103.4 J/g). This is the closest value to the value of the pure LDPE. Thus, this material is more difficult to melt than the other composites. The melting conditions are close to the melting conditions of the pure LDPE film. Therefore, comparing the melting process of the LDPE film with the melting process of the LDPE/CS_BO10 film, no significant differences are introduced by the CS addition.

### 2.5. SEM Morphology

**Figure 5:**
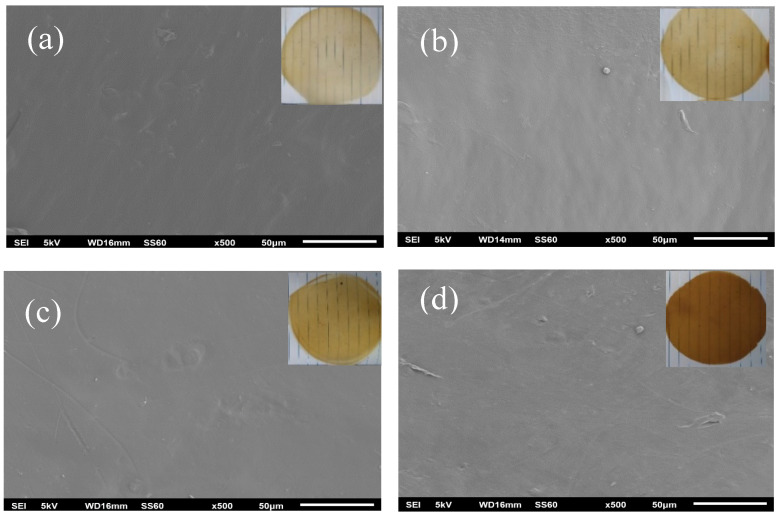
Morphology of (**a**) LDPE/CS_BO5, (**b**) LDPE/CS_BO10, (**c**) LDPE/CS_BO20, and (**d**) LDPE/CS_BO30 films. In the upper right part of each SEM image the photos of corresponding films.

The surface morphology of all the LDPE/CS_BO films was analyzed using a scanning electron microscopy. Figure 5 shows the SEM images of all the LDPE/CS_BO films under ×500 magnification. It is obvious from Figure 5 that the folds and the wrinkles are much more visible on the SEM images (a) and (b) (LDPE/CS_BO5, LDPE/CS_BO10 films) than on the SEM images (c) and (d) (LDPE/CS_BO20 and LDPE/CS_BO30 films). Thus, it could be stated that there are differences on the surfaces of the obtained LDPE/CS_BO films.

### 2.6. Tensile Measurements

**Figure 6:**
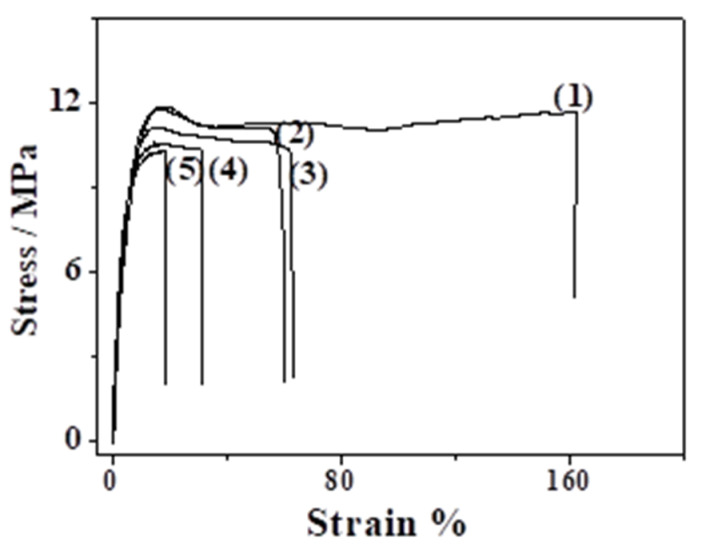
Strain–stress curves of (1) LDPE, (2) LDPE/CS_BO5, (3) LDPE/CS_BO10, (4) LDPE/CS_BO20, and (5) LDPE/CS_BO30.

Table 1 tabulates the mean values and the standard deviation of the Young’s Modulus (E), the tensile strength (σ_uts_), and the elongation at break (ε_b_), which was calculated based on the strain–stress curves (see Figure 6).

By increasing the CS_BO content in the LDPE/CS_BO films, the Young’s Modulus (Ε) increases. This behavior is typical for thermoplastic materials blended with brittle materials such as CS [8,30]. At the same time, by increasing the CS_BO content, the tensile strength decreases, and the % elongation at break decreases further. The increase of the brittle material content such as CS, results to a decrease of ductility [30].

The decrease of tensile strength and of the elongation at break values by increasing the CS concentration was also observed in several previous studies studies [10,11,13]. In these studies [10,11,13], the CS concentrations range from 1 to 10%. Recently, Kusumastuti et al. [9] prepared LDPE/CS blends with CS concentration up to 40 wt%. This became feasible by adding maleic anhydride (MA) and tert-butyl peroxybenzoate (TBPB) as a compatibilizer and initiator, respectively. The obtained LDPE/CS films exhibited better tensile stress and strength values compared to the values of pure LDPE films. In this work, the CS concentration range from 5 to 30 wt%. and a significant increase in tensile stress values recorded by increasing the CS concentration. Thus, the mechanical properties reported in this paper are similar to those reported recently by Kusumastuti et al. [9]. This fact indicates the successful use of the BO as a compatibilizer and implies the good dispersion of the CS_BO blend in the LDPE matrix. The homogeneous blending of the CS_BO with the LDPE chains is also supported by the SEM images of such films, where a good dispersion of the CS_BO blend in the LDPE chains was recorded. This homogeneity enhances the tensile stress values of all the obtained LDPE/CS_BO films.

**Figure 7:**
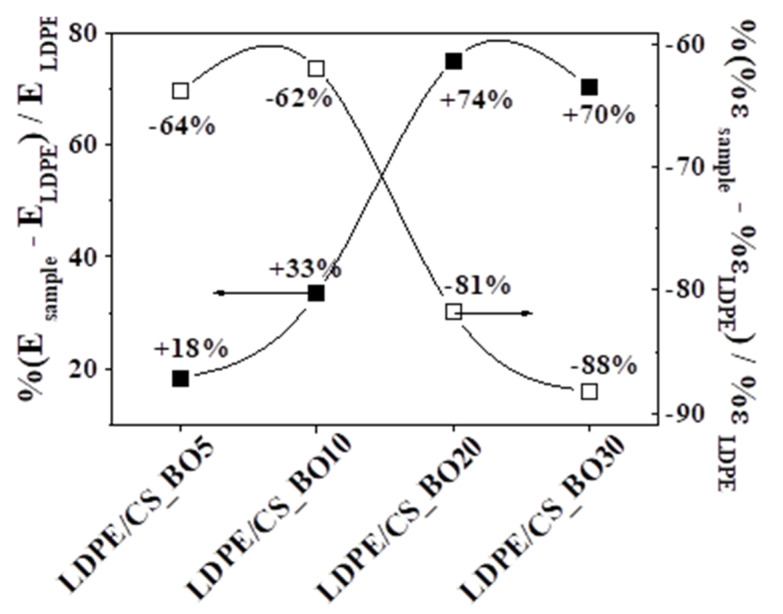
**%** variation of mean values of the Young’s Modulus–E values, and of the mean values of the %elongation at break–%ε values for all tested the LDPE/CS_BO active packaging films.

Figure 7 presents the percentage of variation of the Young’s Modulus (E) and the % elongation at break (ε) values of each sample vs. the corresponding values of the LDPE. Figure 4 indicates the 10% CS_BO loading is the optimum loading to achieve a significant increase of endurance in tensile stress without a significant decrease in the tensile strain of the same property. As a conclusion from the tensile measurements, we could say that LDPE/CS_BO10 sample exhibited the optimum tensile properties.

### 2.7. Water Sorption

The calculated % water sorption values of all the obtained composite LDPE/CS_BO films as well as of the LDPE film are listed in Table 2 [9]. As the CS_BO content increases, the % water sorption increases too. Although the increase of the water sorption values should be expected in the case of a hydrophilic biopolymer addition (i.e., CS) to LDPE, it must be noted that the values reported in this work are lower than the water sorption values reported elsewhere [27] and close to the low water sorption values reported recently by Kusumastuti et al. [9]. This result indicates that the hydrophobic modification process which took place due to the development of the CS_BO blend led to the production of final films with lower water uptake values. To conclude, the water sorption measurements show that LDPE/CS_BO5 and LDPE/CS_BO10 samples exhibited the lowest water sorption values.

### 2.8. Water Vapor Transmission Rate (WVTR)

**Figure 8:**
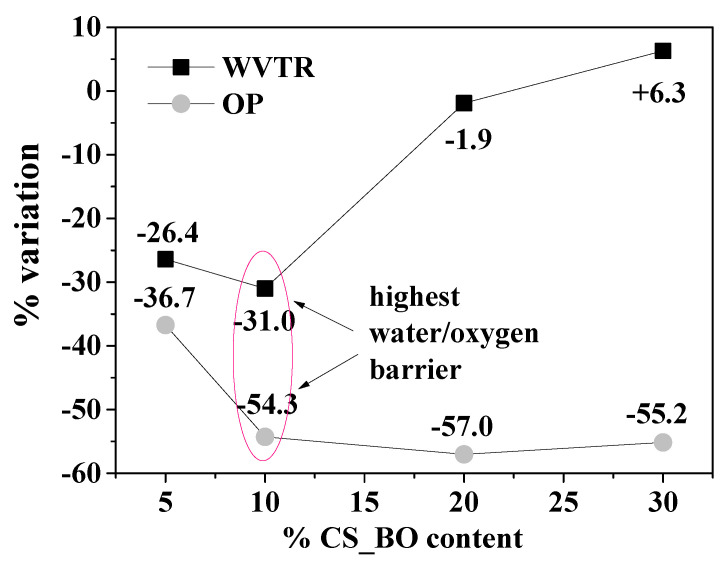
% Water Vapor Transmission Rate (WVTR) and Oxygen Permeability (OP) mean value variation of each LDPE/CS_BO composite sample as compared to the mean values of the WVTR and OP of pure LDPE film.

The calculated WVTR values for all the LDPE/CS_BO active films and for the pure LDPE films are listed in Table 2. The obtained WVTR values of this work cannot be compared with corresponding values reported in literature because the measuring technique followed in this work was developed by our lab-team and is not a regularly used method. Thus, the WVTR results are useful only for comparisons between the tested samples of this work. Figure 8 depicts the % variation of WVTR values compared to the WVTR value of the original LDPE film. 

The WVTR values of the LDPE/CS_BO5 and the LDPE/CS_BO10 samples are lower than the WVTR value of the pure LDPE film at around 26.4% and 31.0%, respectively. On the contrary, for the LDPE/CS_BO20 and the LDPE/CS_30 samples, the respective WVTR values are higher than the WVTR value of the pure LDPE film. In all previous works [8,10,11,13], the obtained LDPE/CS films lost their excellent water vapor transmission properties as compared to the pure LDPE film. The hygroscopic CS acts as a water passageway into the LDPE film matrix. This fact promotes significantly film’s WVTR properties [10,11,13]. It is the first time where a decrease in WVTR values by adding CS in LDPE matrix is reported. This behavior must be attributed to the hydrophobic nature of the used CS_BO blend. Moreover, the WVTR values and the water sorption values exhibited a similar trend. According to the water vapor transmission rate measurements, the LDPE/CS_BO10 sample exhibited the lowest WVTR value.

### 2.9. Oxygen Permeability (OP)

**Figure 9:**
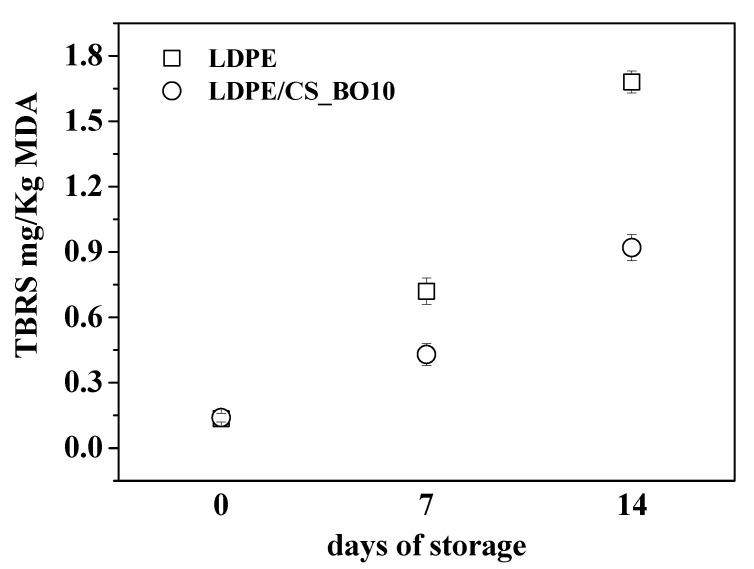
TBARS values of chicken breast fillets vacuum packaged with LDPE, and LDPE/CS_BO10 film after 0, 7, and 14 days of storage at 4 °C.

It is obvious from the OP values, which are listed in Table 2, that the incorporation of the CS_BO blend with the LDPE matrix increases the oxygen barrier of all the obtained LDPE/CS_BO composite films compared to the OP values of the pure LDPE film. This result is similar with the results reported in other previous publications [11,13] where the CS addition improves significantly the oxygen barrier performance of the LDPE/CS films because of the presence of polar interactions in its structure [13]. The Figure 9 plots are present the % variation of the OP values of each sample vs. the OP value of the pure LDPE film. Thus, the OP values of the LDPE/CS_BO5, LDPE/CS_BO10, LDPE/CS_BO20, and LDPE/CS_BO30 films are lower than the OP value of the pure LDPE film at around 36.7%, 54.3%, 57.0% and 55.2%, respectively. The LDPE/CS_BO10 sample exhibits the highest water and oxygen barrier values. 

### 2.10. Overall Migration Rate 

All the obtained LDPE/CS_BO samples exhibited higher values than the corresponding values of the “blank” LDPE sample. By increasing the CS concentration, the OMR values of the LDPE/CS_BO films increased. This trend is also observed by Reesha et al. [11]. Nevertheless, the overall migration values (OMR) of all the LDPE/CS_BO films are found to be lower than the maximum permissible limit of 60 mg/L (see Table 2). The lowest OMR values are obtained for LDPE/CS_BO5 and LDPE/CS_BO10 films. Thus, such films could be more suitable for food packaging applications. 

### 2.11. Antioxidant Activity

By increasing the CS_BO content the antioxidant activity values of the LDPE/CS_BO active films also increased (see Table 2). The estimated values are 6.4% for the LDPE/CS_BO5, 12.8% for the LDPE/CS_BO10, 22.4% for the LDPE/CS_BO20, and 34.6% for the LDPE/CS_BO30 sample.

### 2.12. Lipid Oxidation

Figure 9 plots present the calculated TBARS values of chicken breast fillets which were packaged with the most active film, i.e., the LDPE/CS_BO10 film, as well as with the “blank” LDPE film. The storage period was seven (7) and fourteen (14) days and the storage temperature was 4 °C. The initial TBARS values of chicken breast fillets were estimated at around 0.14 mg MDA/kg. Such values are consistent with others reported previously [31,32]. 

The TBARS values of chicken breast fillets packaged with “control” LDPE film, were measured after storage under vacuum, at 4 °C, for seven and fourteen days. These values were found 0.72 and 1.68 mg MDA/Kg meat, respectively. The TBARS values for the same storage conditions, packaged with LDPE/CS_BO10 active film, were found 0.43 and 0.92 mg MDA/Kg meat, respectively. Chicken breast fillet, which was packaged with “control” LDPE film and stored for fourteen days exhibited, had TBARS values limited below 2 mg/kg. This value is the threshold at which an odor becomes noticeable because significant lipid oxidation occurs [33]. The TBARS value of chicken breast fillet, which was packaged and stored for fourteen days, but with the LDPE/CS_BO10 active packaging film was well below the 2 mg/kg threshold. Moreover, the TBARS values for chicken breast fillet, which was packaged with LDPE/CS_BO10 film and stored for seven and after fourteen days, was found to be lower, at around 41% and 45%, respectively, compared to the TBARS values for chicken breast fillet, which was packaged with “control” LDPE film and stored for the same time periods. Thus, the antioxidant activity and the improved barrier properties of LDPE/CS_BO10 film extends the storage period of chicken breast fillets which were packaged at 4 °C under vacuum. 

### 2.13. Statistical Analysis of the Experimental Data

The used confidence interval for all tests is the most common value of C.I. = 95%. Thus, the value of the statistical significance level is *p* = 0.05. The influence of the different compositions on the final product properties was statistically confirmed starting with the hypothesis H0: (Mean values of this property could be assumed as equal for all different compositions). This was performed for supporting the hypothesis that every parameter has a statistically different mean value considering samples with different CS_BO compositions. We used the nonparametric Kruskal–Wallis method, and the results are presented in Table 3. Comparing the Sig. values from Table 3 with the significance level *p* = 0.05, it is obvious that in all cases and for all parameters, the mean values are statistically different. According to the developed empirical equation that is reported in the in the literature [34] and concerns the calculation of an empirical factor, the so-called “inequality assurance” (IA), it is obvious from Table 4 that in all cases the inequality of mean values is statistically assured strongly (IA >= 88%). The IA factor represents the percentage of the deviation toward zero (0) of the Sig. value from the significance level value (*p*). 

## 3. Materials and Methods

### 3.1. Materials

The LDPE was supplied by Aldrich, Darmstadt, Germany (cat. no. 428027), with melt flow index of 1.5 g/10 min (190 °C/2.16 kg) and with density d = 0.922 g cm^−3^. The CS with medium molecular weight and with deacetylation degree of 90% was supplied from Flurochem, Hadfield, Derbyshire, United Kingdom (cat. no. FCB051814). The basil essential oil (BO) was purchased from Esperis spa., and according to its safety data sheets, the % mass composition was 70–80% estragole, 7.5–10% linalool, 1–3% eucalyptol, 0.5–1.0% eugenol, and 0.5–1.0% D-limonene.

### 3.2. Preparation Methods

#### 3.2.1. Preparation of CS_BO Hybrid Blend

Modified CS_BO hybrid blend was prepared via a green evaporation method. This method is similar to a method previously reported in the literature for the modification of clays with thyme, oregano, and basil oil [25]. This method blended successfully the CS with the most volatile fraction of the EO.

**Figure 10:**
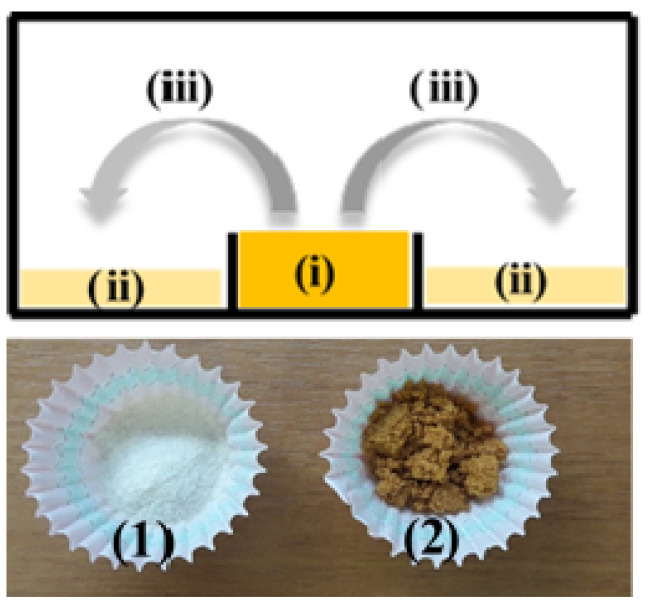
Upper part: Schematic representation of the green evaporation/adsorption process applied for the preparation of BO_CS nanostructure, (i) glass beaker with BO, (ii) spread CS at the bottom of the aluminum beaker, (iii) process of BO evaporation and adsorption into CS. Down part: (1) Image of raw CS powder and (2) modified CS_BO nanostructure.

The preparation method of this study exploits the advantage of the BO evaporation ability and the CS adsorption capacity to prepare an improved CS_BO blend, which can easily be used as a masterbatch in the packaging industry. An amount of 10 g of CS (see Figure 10(ii)) was spread in an aluminum beaker. In the middle of this aluminum beaker, a smaller quartz beaker was placed and filled with 10 g of BO (see Figure 10(i)). The whole “apparatus” was sealed and put in an oven at 120 °C for 24 h. Under these conditions, the most volatile BO components were evaporated and adsorbed into the CS (see Figure 10). Following this green method, the use of organic solvents was avoided, and the CS adsorbed as much BO as it could. Moreover, the produced CS_BO hybrid is in powder form (see Figure 10) and can be accurately weighed and used as a masterbatch in industry and incorporated in the extruder to produce the packaging film. The obtained BO loading on CS was calculated gravimetrically and was approx. 18.5 wt%.

#### 3.2.2. Preparation of LDPE/CS_BO Active Films

The LDPE/CS_BO films were produced via a melt mixing process using a minilab twin co-rotating extruder. The uniform operating temperature of the melt process was 140 °C and the screw speed was 100 rpm. The total processing time was 5 min. The nominal composition of the used CS_BO blend was fixed at 5, 10, 20, and 30 wt%.

The produced melted material was cut into small granules using a granulated machine. The final films were produced by a hot-pressing process. Approximately, 1 g of granules were pressed at 110 °C under 2.0 MPa constant pressure for 3 min, using a hydraulic press with heated platens. Finally, a sample of pure LDPE is also extruded and used as reference sample for comparison reasons. In Table 4 is presented the code names of all the produced samples, the used amounts of the LDPE and CS_BO material, and the extruder processing conditions for the preparation of all the LDPE/CS_BO active films.

### 3.3. XRD Analysis

The morphological characteristics of the CS_BO and neat CS powders, and of all the produced films were investigated via XRD measurements using a Brüker D8 Advance X-ray diffractometer (Brüker, Analytical Instruments, S.A. Athens, Greece) equipped with a LINXEYE XE High-Resolution Energy-Dispersive detector. Typical scanning parameters were set as follows: Two theta range 2–30° for powder as well as for film samples; increment 0.03°; PSD 0.764.

### 3.4. FTIR Spectrometry

The chemical structure of the CS_BO and neat CS powders and of the produced films (pure LDPE and LDPE/CS_BO composite films) was investigated by IR spectra measurements. Infrared (FTIR) spectra, which were the average of 32 scans at 2 cm^−1^ resolution, measured with an FT/IR-6000 JASCO Fourier transform spectrometer (JASCO, Interlab, S.A., Athens, Greece) in the frequency range 4000–400 cm^−1^.

### 3.5. Thermal Studies TG-DTA/DSC

Thermogravimetric (TGA) and differential thermal analysis (DTA) were performed on pure CS, modified CS_BO hybrid blend, and all the produced LDPE/CS_BO samples. Measurements performed by a Perkin-Elmer Pyris Diamond TGA/DTA instrument (Interlab, S.A., Athens, Greece). Samples of approximately 5 mg were heated under an N_2_ flow, from 25 to 700 ℃, with a temperature increasing rate of 5 K/min. The thermal behavior of the obtained LDPE/CS_BO film and of the reference LDPE film was also studied using a DSC214 Polyma Differential Scanning Calorimeter (NETZSCH manufacturer, Selb, Germany). Samples between 1.2–3.3 mg were tested in the temperature range from −30 to 200 °C at a heating rate of 10 K/min under a nitrogen atmosphere. 

### 3.6. Scanning Electron Microscopy (SEM) of LDPE/CS_BO Blends

Scanning electron microscopy (SEM) images were obtained using a JEOL JSM-6510 LV SEM Microscope (JEOL Ltd., Tokyo, Japan) equipped with an X-Act EDS-detector by Oxford Instruments, Abingdon, Oxfordshire, UK (an acceleration voltage of 5 kV was applied). The specimens were sputtered with an Au-Pd thin film (4–8 nm) using a mini sputter coater SC7620 from Quorum Technologies LTD (Kent, UK).

### 3.7. Tensile Properties

Tensile measurements were carried out on all the produced LDPE/CS_BO films and were compared with the relevant measurements of the “blank” LDPE film. The measurement procedure was according to the ASTM D638 method using a Simantzü AX-G 5kNt instrument (Simantzu. Asteriadis, S.A., Athens, Greece). Three to five samples of each film were tested at a crosshead speed of 2 mm/min. The samples were dumbbell-shaped with gauge dimensions of 10 mm × 3 mm × 0.22 mm. Force (N) and displacement (mm) were recorded during the test. 

### 3.8. Water Sorption

Water sorption measurements were carried out following a methodology, which was described previously [35,36]. The obtained films were cut into small pieces (12 mm × 12 mm), desiccated overnight under vacuum, and weighed to determine their dry mass. The weighed films were placed in closed beakers containing 30 mL of water (pH = 7) and stored at T = 25 °C. The sorption plots were developed by the periodical weighting of the samples until sorption equilibrium was reached. The Equation (1) used for such calculations was:

W.S. (%) = (m_Wet_ × m_Dry_)/m_Dry_ × 100 (1)

where m_Wet_ and m_Dry_ are the weight of the wet and dry film, respectively, and W.S. is the Water sorption.

### 3.9. Water Vapor Transmission Rate (WVTR)

WVTR of all the obtained films was determined. Experimental conditions were fixed at 38 °C and 50% RH according to the ASTM E96/E 96M-05 method using a handmade apparatus according to previous *reports* [23,37,38]. Circular disk films with 10 μm average thickness and 2.5 cm diameter were placed on the top of plexiglass cylindrical tubes with closed bottoms as was described in detail in the above mentioned publications. These cylindrical tubes contained dried silica gel inside, and the films were sealed with a rubber O-ring. The devices were placed in a glass desiccator which contains 200 mL of saturated magnesium nitrate solution (50% RH). Each film was weighed before and after the measurement to ensure that no water adsorption or dissolution phenomena occurred during the experiment. Tested cylinders were weighed periodically for 24 h and the WVP was calculated according to Equation (2):

WVP = (ΔG/t)/A(2)

where ΔG is the weight that the tested cylinders gained in g, t is the time in hours, and A is the permeated area of the film. The ΔG/t term is estimated by the slope of the fitted straight line over the ΔG = f(t) plot scattering.

### 3.10. Oxygen Permeability (OP)

The oxygen transition rate (OTR) of all the obtained LDPE/CS_BO and neat LDPE films was measured using an oxygen permeation analyzer (8001, Systech Illinois Instruments Co., Johnsburg, IL, USA). All samples were tested at 23 °C and 0% RH according to the ASTM D 3985 method. OTR values were expressed at cc O_2_/m^2^/day. The OP values of the tested samples were calculated by multiplying the OTR values with an average film thickness of around 350–400 μm. The mean OTR value for each kind of film resulted from the measurements of three samples.

### 3.11. Overall Migration Test

The overall migration measurements of all the LDPE/CS_BO films were carried out according to the USFDA 176:170 test procedure [39]. Samples of each film were cut to produce specimens of 1 dm^2^ and immersed in a glass tube with 250 mL of stimulating solvent (water) at 49 °C for 24 h. After exposure to the atmosphere for a specified duration, the film was dried, and the solvent evaporated. The residues were weighed, and the overall migration residue (OMR) values were calculated according to the Equation (3):

OMR in mg/L = (mass of residue (mg) × 1000)*/*(Volume of stimulant (mL)) (3)

### 3.12. Antioxidant Activity

The antioxidant activity of films evaluated according to a methodology which was described previously [40], but with small modifications. An amount of 500 mg of small pieces (approximately 3 mm × 3 mm) of each film was used. The sample was placed in a dark-colored glass bottle with a plastic screw cap and filled with 10 mL ethanolic solution of DPPH, 50 ppm (mg/L). After incubation at 25 °C for 24 h in darkness, the % antioxidant activity values of the films were calculated according to the Equation (4):

% Antioxidant activity = (Abs_control_ × Abs_sample_)/Abs_control_) × 100 (4)

### 3.13. Lipid Oxidation Test

**Figure 11:**
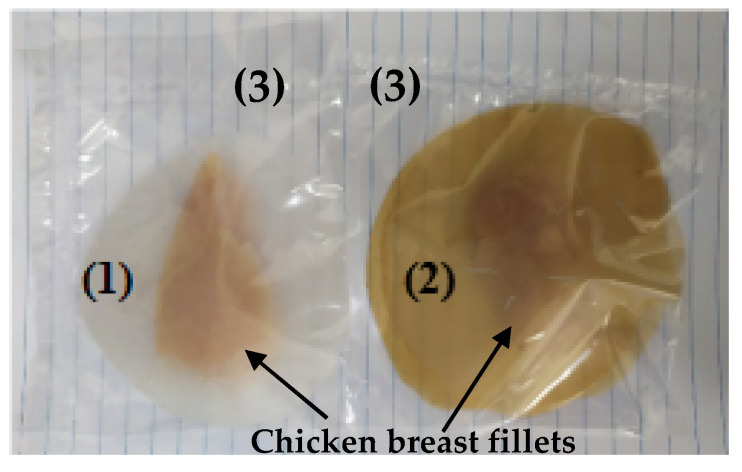
Chicken breast fillets vacuum packaged with (1) “control” LDPE film, (2) LDPE/CS_BO10 film, and (3) package foil.

The lipid oxidation test was carried out according to a methodology which was described previously [31] but with small modifications. Briefly, for the packaging of each piece of freshly skinned and deboned chicken breasts (around 20 g each), two disk-shaped films with a 10 cm diameter and a 0.06 mm average thickness were used. The chosen film for lipid oxidation experiments was the LDPE/CS_BO10. This film exhibited the highest water/oxygen barrier, improved tensile properties, and improved antioxidant activity. For “blank” measurements, two neat LDPE disk-films were used. The two disk-shaped samples (10 cm diameter, 100 μm thickness) of each film were put inside a commercial polyethylene (PE) packaging bag, (see Figure 11). For reliable results, three samples of each film were tested, and the measurements were analyzed statistically. To avoid cross-contamination, all used utensils, including the disk-shaped films and PE packaging bags were sanitized with ethanol. Τhe samples were packed under vacuum using a vacuum sealer machine SFS 120 A1 and stored at temperature of 4 °C. A batch of samples was stored for seven days while another batch was stored for fourteen days. All samples were analyzed following the thiobarbituric acid reactive substance (TBARS) method. The determination of their lipid oxidation degree was carried out according to a methodology which was described previously [34,41]. 

### 3.14. Statistical Analysis

All measurements were carried out at least in triplicate for each sample. The statistical analysis was performed using the Statistical Package SPSS 20 for windows (SPSS Inc., Chicago, IL, USA). The mean values and standard deviation are presented above in Table 1 and Table 2. 

## 4. Conclusions

In advance it could be stated that the modification of the CS with BO molecules via a “green” adsorption/desorption process lead successfully to a hydrophobic CS_BO bioactive blend that can be used as a masterbatch in extrusion molding processes. The developed LDPE/CS_BO active packaging films exhibited enhanced tensile, barrier, and antioxidant properties. In advance, the decrease of the WVTR values by increasing the CS_BO content is reported for the first time for such LDPE/CS composite films. The most promising film in this work was the composite LDPE/CS_BO10. This film shows an enhanced tensile stress value without a significant decrease to the %elongation at break value, the highest water/oxygen barrier properties as they compared to the water/oxygen barrier properties of the other samples and of the pure LDPE film. Moreover, the LDPE/CS_BO10 film exhibits a melting behavior close to this of the initial pure LDPE, and a significant antioxidant activity which was higher than the relevant of the pure LDPE films. Chicken breast fillets packaged with this film and stored under vacuum at temperature 4 °C for 14 days, exhibit around 45% lower TBARS value than the corresponding value of fillets packaged with pure LDPE film and stored under the same conditions. This material could be a promising, active packaging film. This study is innovative, promising, and could be used as a guide for the incorporation of other essential oils in such LDPE/CS films.

## Figures and Tables

**Table 1:** Melting point temperature, fusion enthalpy values among with Modulus of elasticity (E), tensile strength (σ_uts_), and % elongation at break (ε_b_) of all tested LDPE/CS_BO films.

Code Name	Melting Point T (°C)	Fusion Enthalpy ΔΗ_f_ (J/g)	Young’s Modulus E (St. Dev.) (MPa)	Tensile Strength σ (St. Dev.) (MPa)	% Elongation at Break (ε) (St. Dev.)
LDPE	110.8	113.5	203.3 ± 42.4	13.7 ± 1.3	161.0 ± 72.0
LDPE/CS_BO5	108.4	97.2	240.7 ± 39.6	11.6 ± 0.7	58.3 ± 10.6
LDPE/CS_BO10	109.3	103.4	271.3 ± 47.1	9.7 ± 1.5	61.3 ± 25.0
LDPE/CS_BO20	107.8	81.6	355.7 ± 49.1	9.5 ± 0.9	29.3 ± 9.4
LDPE/CS_BO30	108.5	79.1	346.0 ± 57.4	9.3 ± 0.6	19.0 ± 11.2

**Table 2:** Water vapor transmission rate (WVTR), water sorption, oxygen permeability (OP), total migration, and antioxidant activity values of all tested LDPE/CS_BO active films.

Code Name	WVTR (St. Dev.) (g/m^2^·day)	% Water Sorption (St. Dev.)	OP (St. Dev.) cm^3^·mm/m^2^·day	Total Migration (St. Dev.) (mg/L)	Antioxidant Activity after 24 h (St. Dev.)
LDPE	19.49 ± 1.5	0.00 ± 0.00	182.4 ± 3.4	12.4 ± 0.1	-
LDPE/CS_BO5	14.35 ± 1.2	0.12 ± 0.05	115.4 ± 2.8	15.5 ± 0.1	6.4 ± 0.9
LDPE/CS_BO10	13.45 ± 1.8	0.17 ± 0.05	83.3 ± 3.5	17.6 ± 0.1	12.8 ± 1.2
LDPE/CS_BO20	19.12 ± 1.5	0.27 ± 0.05	78.4 ± 2.5	25.3 ± 0.1	22.4 ± 1.4
LDPE/CS_BO30	20.71 ± 2.2	1.15 ± 0.60	81.8 ± 5.4	45.4 ± 0.1	34.6 ± 1.5

**Table 3:** Mean values inequality test of modulus of elasticity (E), tensile strength (σ_uts_), % elongation at break (ε_b_), water vapor permeability WVP, % water sorption, oxygen permeability (OP), total migration, % antioxidant activity after 24 h, and TBARS values of all produced films.

	Sig.	IA
E	0.002	96
σ_uts_	0.006	88
%ε	0.002	96
WVP	0.003	94
% water sorption	0.005	90
OP	0.004	92
Total migration	0.002	96
TBARS	0.003	94
% Antioxidant activity after 24 h	0.000	100

**Table 4:** Code names and amounts of the used LDPE and CS_BO. Extrusion processing conditions for all the prepared active films.

Code Name	LDPE (g)	CS_BO (g)	Extrusion Temperature (°C)	Extrusion Rotation Speed (rpm)	Extrusion Total Processing Time (min)
LDPE	5.00	-	140	100	5
LDPE/CS_BO5	4.75	0.25	140	100	5
LDPE/CS_BO10	4.50	0.50	140	100	5
LDPE/CS_BO20	3.00	1.00	140	100	5
LDPE/CS_BO30	3.50	1.50	140	100	5

## Data Availability

Not applicable.

## Sample Availability

Samples of the compounds are available from the corresponding authors Giannakas A.E. and Salmas C.E.
